# Partnering with periodontal patients and care providers to establish research priorities for patient engagement in specialized periodontal care: A study protocol

**DOI:** 10.1371/journal.pone.0319841

**Published:** 2025-03-25

**Authors:** Arnaldo Perez, Manuel Lagravere, Cristine Miron Stefani, Ava Nasr Esfahani, Geoff Ball, Monica Gibson

**Affiliations:** 1 School of Dentistry, Faculty of Medicine and Dentistry, University of Alberta, Edmonton, Alberta, Canada; 2 Department of Dentistry, Health Sciences School, University of Brasilia, Brasilia, Brazil; 3 Department of Pediatrics, Faculty of Medicine and Dentistry, University of Alberta, Edmonton, Alberta, Canada; 4 School of Dentistry, Indiana University, Indianapolis, Indiana, United States of America; University of Minnesota School of Dentistry, UNITED STATES OF AMERICA

## Abstract

**Introduction:**

Periodontitis is highly prevalent and disproportionately affects vulnerable populations, including older adults, racial and ethnic minorities, and low-income individuals. While periodontal therapies are largely effective, patient engagement in periodontal care is problematic. The study describes in this protocol aims to identify the top ten research priorities or uncertainties for specialized periodontal care (SPC) that are most important to periodontal patients and care providers.

**Methods:**

The James Lind Alliance approach will guide the priority-setting partnership (PSP), which involves several steps: forming a PSP steering committee, gathering potential research uncertainties, summarizing the research uncertainties, verifying unanswered uncertainties, completing an interim priority setting survey, and facilitating a priority setting workshop. Study participants will be periodontal patients (n ~ 150) and care providers (n ~ 120), including general dentists, periodontists, and dental hygienists in Alberta, Canada. A steering committee representing the four stakeholder groups will oversee the study. Data on uncertainties from these groups will be gathered through two online surveys and focus groups. Demographic data (*e.g.,* age, sex) will be collected to describe participants and ensure representation of all stakeholder groups. Uncertainties submitted by participants will be evaluated against the existing evidence gathered through a scoping review to determine if they have already been addressed. Unanswered uncertainties will be taken to a workshop where participants (n ~ 20) representing all the stakeholder groups will set the top ten research priorities. Data analysis will include descriptive statistics and content analysis. The study is expected to conclude in August 2026.

**Conclusion:**

Study findings will be disseminated to raise awareness among researchers and funders on research priorities that matter most to patients and dental care providers regarding patient engagement in SPC.

## Introduction

Periodontitis is highly prevalent, has adverse consequences for individuals and healthcare systems, and disproportionally affects vulnerable populations. Periodontitis is the sixth most prevalent disease worldwide [1]. Nearly half of adults in the United States have periodontitis [2], and one-quarter of adults in Canada suffer from severe periodontitis [3]. This condition has adverse, long-lasting consequences for individuals, including tooth loss, impaired masticatory function, nutritional compromise, altered speech, low self-esteem, and sub-optimal quality of life [4–6]. Periodontitis has been associated with non-communicable chronic conditions such as cardiovascular disease and diabetes mellitus [7]. Periodontitis also imposes a substantial financial burden on individuals and healthcare systems based on both direct and indirect costs [8]. Several groups are particularly impacted by this condition, including older adults, low-income individuals, racial and ethnic minorities, and people with disabilities. These groups also experience elevated levels of untreated dental conditions [9,10].

Available therapies, both non-surgical and surgical, are largely effective in managing periodontitis. Regular patient education emphasizing the importance and proper implementation of home care of biofilm removal has been effective in preventing and managing periodontitis as evidenced by improvements in plaque index and gingival index [11]. Research has consistently reported positive clinical outcomes (e.g., pocket probing reduction, clinical attachment level gain) when local and systemic antibiotics are used as adjunctive therapy to scaling and root planing in the treatment of moderate to severe periodontitis [12,13]. Traditional periodontal surgery effectively reduces periodontal probing depth and enhances periodontal architecture, while emerging approaches hold promise for halting periodontitis and promoting periodontium regeneration [14]. Periodontal treatment can also enhance non-clinical outcomes, such as oral health-related quality of life [15].

Patients can benefit from periodontal therapies if they optimally engage in periodontal services [13,16]. Patient engagement in health services broadly refers to the process of seeking and utilizing these services, including whether, how, why, and for how long the services are used [17]. Active engagement of patients has been found to improve health behaviors, clinical outcomes, quality of life, quality and efficiency of services, and patient empowerment [18,19]. Research has shown that adherence to periodontal treatment improves outcomes, including a reduction in biofilm and gingival bleeding rates [16,20]. Negative outcomes, such as increased probing depth and tooth loss, have been associated with poor adherence to clinical appointments, with adherence to periodontal supportive therapy ranging from 2% to 64% [21,22].

The available literature on patient engagement in periodontal care is limited, outdated, and inconclusive. Most original research on this topic has used quantitative approaches and focused on referral and adherence to periodontal care. Research on referral has largely centered on demographic predictors related to patients (e.g., socioeconomic status, type of dental insurance) and referral providers (e.g., age, gender, location), which falls short of revealing the complexity of the referral process [23,24]. Similarly, most research on patient adherence has focused on rates and predictors of appointment-keeping during the maintenance phase in general practice, such as age, smoking status, and disease severity [25,26]. Predictors and factors influencing patient involvement during the diagnosis and active treatment phases in settings such as dental school clinics have been relatively understudied. Three recent reviews indicated that the majority of studies on periodontal referral and adherence were published in 2010 or earlier [16,22,24]. Although limited in scope and depth, these reviews concluded that the available evidence is insufficient for understanding and improving patient engagement in periodontal care.

Currently, there is a need for expanding and enhancing the scope and relevance of research on patient engagement in periodontal care. To address this need, the involvement of healthcare stakeholders, especially patients and care providers, as equal partners is paramount [27,28]. This study aims to identify the top ten research priorities or uncertainties for patient engagement in specialized periodontal care (SPC) by gathering insights from periodontal patients and care providers, including general dentists, periodontists, and dental hygienists. This study will seek to answer the following questions: What are the top ten research priorities for periodontal patients and care providers regarding patient engagement in SPC? What are the issues regarding patient engagement in SPC that matter most to periodontal patients and care providers? The study findings will be important for guiding future research directions and optimizing the allocation of financial resources through both public and private funding.

## Materials and methods

### Design

**Fig 1:**
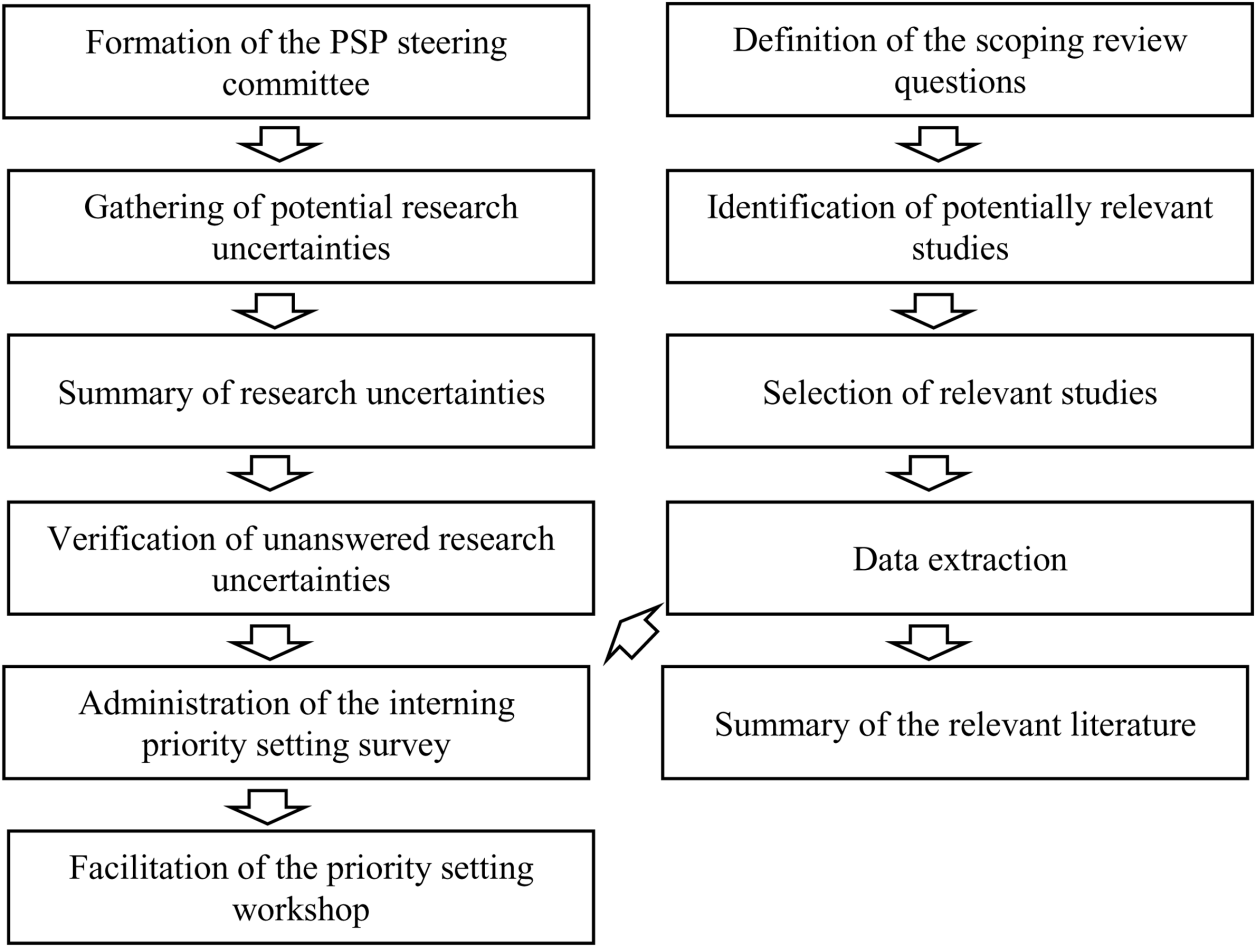
Flow chart of the PSP for patient engagement in SPC, including a scoping review.

The study design will be guided by the James Lind Alliance (JLA) approach, which includes the following steps: *(1)* forming a priority setting partnership (PSP) steering committee, *(2)* gathering potential research uncertainties, *(3)* summarizing the research uncertainties, *(4)* verifying unanswered uncertainties, *(5)* completing an interim priority setting survey, and *(6)* facilitating a priority setting workshop [27]. As a partnership-driven approach [29], the James Lind Alliance (JLA) is well-suited to identify relevant research priorities for both practice and science through collaboration with healthcare stakeholders, particularly patients, caregivers, and care providers. In recent years, this inclusive approach has been successfully used to set research priorities for several chronic conditions, including diabetes [30], dementia [31], Parkinson [32], chronic kidney [33], and eating disorders [34]. The study is expected to conclude in August 2026. Fig 1 illustrates all the steps of the PSP, including the scoping review that will support this process.

### Research setting and participants

The study is expected to be conducted over a two-year period, from September 2, 2024 to August 31, 2026, and will involve periodontal patients and care providers in Alberta, Canada. Former and active periodontal patients, general dentists, periodontists, and dental hygienists practicing in Alberta for at least three years will be eligible to participate. Sample size estimates are not calculated for JLA-PSP projects [27]; however, based on previous similar studies [33,35,36], we estimate the enrollment of ~ 150 patients, ~ 60 general dentists, ~ 20 periodontists, and ~ 30 dental hygienists. Several recruitment strategies will be employed. Patients will be approached in person in clinical settings and contacted using clinic registries. They will also be informed about the study through local advertisements (e.g., posters), social media, and grassroots agencies. These strategies will help reach groups that have been historically marginalized and disproportionally affected by periodontal diseases. Care providers will be reached in person and through professional organizations’ communication channels (e.g., email lists, newsletters), social media (e.g., specific websites and platforms), local advertisements, and individual networks. Patients and care providers will be encouraged to invite other eligible participants.

### Step 1. Forming a PSP steering committee (SC)

This committee will oversee all study activities to ensure adherence to the JLA process. Representation of all stakeholder groups will be achieved by recruiting 2 patients, 2 general dentists, 2 periodontists, and 2 dental hygienists. SC members will be offered tokens of appreciation (e.g., $25 gift card) for their participation in the meetings (n = 6) over the course of the project. Three research team members will join the SC. Meetings will take place every three months via Zoom, lasting approximately two hours. Minutes will be taken to document decisions, including any remedial actions needed to address issues related to study implementation, ensure adherence to the JLA process, and track the implementation of suggested changes in subsequent meetings.

### Step 2. Gathering potential research uncertainties through survey and focus groups

An online survey using REDCap will be used to collect uncertainties, concerns, and issues that periodontal patients, general dentists, periodontists, and dental hygienists would like to be answered by research. The survey will be informed by the literature on data collection of uncertainties from stakeholders, following the JLA-PSP approach [27,28]. A total of five open-ended questions will be included, allowing respondents to elaborate on uncertainties and issues that matter to them. For example, respondents will be asked, ‘What questions and concerns would you like researchers to address regarding patient compliance with periodontal treatment?’ There will be no limit on the number of uncertainties respondents can suggest. The online survey will be available until saturation is reached, defined as the point when no substantially new uncertainties are suggested. To maximize participation, several strategies will be implemented. These include offering a $10 gift card as an incentive, clearly communicating the study’s benefits for patients, care providers, health services, and future research in the information letter, and collaborating with community agencies, clinical sites, and professional organizations. Additionally, reminder notifications will be sent to encourage participation throughout the survey period.

Five focus groups with patients and six with different care provider groups (~7 participants in each focus group) will be conducted via Zoom or in-person, depending on participant preferences, and recorded to facilitate data analysis afterwards. These focus groups will explore their perspectives on research priorities and engagement issues. Focus group participants will receive tokens of appreciation (e.g., $25 gift card) for their participation. Interview guides will be informed by previous studies and general guidelines for conducting focus groups [37]. Table 1 presents the main six focus group questions for patients. Similar follow-up questions will be asked for each main question, such as: ‘What aspects worked well or did you like?’ ‘What were the main issues or challenges, if any?’ and ‘What would you like researchers to explore about this particular topic?’ Focus group questions for care providers will vary based on their type and level of involvement in periodontal care. For example, referral providers such as general dentists and dental hygienists will be asked, ‘What has been your experience with referring patients to periodontal care?’ Table 2 outlines the main questions that will be asked to all periodontal care providers.

**Table 1:** Summary of the main focus group questions for patients.

How would you briefly describe your experience with getting and using periodontal care? What was it like for you to be referred to periodontal care? What happens at your first appointment when your periodontist checked your gum health? How was it for you to attend the other appointments? What has it been like to follow your periodontist’s advice? Overall, how would you describe your communication with your periodontist?

**Table 2:** Summary of the main focus group questions for care provider.

Overall, how would you describe patient engagement in periodontal care? How do patients typically engage in the initial assessment of their periodontal status? What have you observed and concluded about patient attendance at periodontal clinical appointments? What have you noticed regarding patient compliance with treatment recommendations across periodontal therapies, including non-surgical, surgical, and supportive treatments? Overall, what communications challenges have you identified when interacting with patients undergoing periodontal care?

The survey and focus group interview guides for each stakeholder group will be pilot tested with similar others using standardized approaches. Data from the survey and focus groups will cover patient engagement (e.g., attendance at clinical appointment, compliance with treatment recommendations) across all critical phases of periodontal care, including referral, initial assessment, active treatment, and maintenance. Demographic information will be obtained to describe participants and ensure that vulnerable populations within the patient group are adequately represented. We anticipate ~ 600 uncertainties upon completion of this step.

### Step 3. Summarizing the research uncertainties

All the uncertainties or questions obtained through the survey and focus groups will be reviewed, sorted, and turned into indicative questions by members of the research team, and will later be discussed with the SC. Reviewing suggested uncertainties will include removing duplicate and similar uncertainties, excluding unrelated uncertainties, combining related uncertainties, and turning complex uncertainties into specific uncertainties. Sorting uncertainties will include coding and grouping them into categories within main engagement issues (e.g., referral, adherence). Lastly, uncertainties will be re-worded, if necessary, into single, indicative questions to improve clarity and understanding. Although unrelated uncertainties will be excluded from this study, they will be considered for future research by the team and shared with the broader research community through a non-original article, such as a letter to the editor or a short communication paper, in a periodontology-related journal. We expect approximately 100 engagement-related uncertainties upon completion of this step.

### Step 4. Verifying unanswered uncertainties

Given that the available literature on patient engagement in SPC has not been comprehensibly summarized and previous reviews have served different purposes, the research team will conduct a scoping review to map the breadth and depth of the research activity on this topic. This review will be used to verify whether the identified uncertainties have been answered and will include a quality assessment of the included studies. While this assessment is optional in scoping review studies, it will be necessary to assess whether the available evidence is credible enough to answer the suggested uncertainties. The quality assessment will be performed using design-specific critical appraisal tools (e.g., for cohort studies) and grading systems [38]. The scoping review will be initiated in step 1 to enable the utilization of the review findings in this step. Arksey & O’Malley’s multi-step framework will guide the scoping review [39], which will be reported according to the PRISMA Extension for Scoping Reviews guidelines [40]. Specifically, this review will characterize the types, sources, quality, and coverage of the available evidence to establish the existing knowledge and gaps in the literature. The assessment of the existing knowledge will be complemented by consulting previous reviews, position statements, and clinical guidelines related to patient engagement in periodontal services, including the referral process. Uncertainties already answered will be excluded, while unanswered or partially answered questions will be considered in the next step. We expect 60 to 70 unanswered or partially answered uncertainties upon completion of this step.

### Step 5. Completing an interim priority setting survey

Respondents of the survey who provided contact information will be invited to complete a second REDCap survey, which will include the remaining uncertainties from Step 4. They will be asked to rank their top ten uncertainties based on perceived relevance for patient engagement in SPC. Uncertainties included in this survey will be organized by engagement issue and randomly order within each issue. This survey will remain available for two months based on times allocated for completion in previous studies [41,42]. Responses from each participant will be recorded as follows: an uncertainty ranked 1 will be given 10 points, an uncertainty ranked 2 will be given 9 points, and so on down to an uncertainty ranked 10, which will be given 1 point. Total points per research uncertainty will be calculated to rank them from highest to lowest across engagement issues. We anticipate approximately thirty uncertainties upon completion of this step.

### Step 6. Facilitating a priority setting workshop

All participants involved in steps 2 and 5 will be invited to participate in an in-person workshop to discuss, refine, and rank the top ten research uncertainties from the priority list generated in the previous step. Potential participants will be informed about the workshop’s purpose, benefits, risks, and their role, as well as the roles of other participants, such as the facilitator and members of the SC. Approximately 20 participants representing all the stakeholder groups are expected to be involved. We do not anticipate excluding any individuals who wish to participate at this stage, as our resources allow us to accommodate additional participants if necessary. Members of the SC will observe the workshop, which is expected to last approximately 2 hours. The workshop will be conducted by a facilitator trained in using the nominal group technique to build consensus [43], and will take place at the School of Dentistry at the University of Alberta. Workshop participants will receive tokens of appreciation (e.g., $25 gift card) for their involvement. The SC will review the top ten research priorities to further improve their wording in order to ensure understanding and facilitate dissemination.

### Data management and analysis

Survey data will be managed using REDCap and exported to Excel for descriptive statistical analysis. Zoom recordings will be automatically transcribed, while professional transcription services will be employed for the in-person focus group recordings. All transcriptions will be reviewed for accuracy before proceeding with data analysis. Inductive, manifest content analysis will be used to analyze qualitative data from the open-ended survey questions and focus groups [44]. These data will be coded by segment of meaning, grouped into potential categories, and checked to assess whether potential categories actually account for the data included within each and the entire data set. Revised categories will then be refined, structured, and re-labeled, if necessary. Representative quotes will be used to support the analysis. Two research team members with formal training in qualitative research will lead the data analysis. The results of their analysis (e.g., developed categories and sub-categories) will be reviewed and discussed with the rest of the research team and presented to the steering committee for further input and discussion. Both quantitative and qualitative data will be kept anonymous and confidential. Quantitative data will be aggregated (e.g., only group-level data will be reported) and qualitative data will be pseudonymized to ensure confidentiality.

### Ethics

Ethics approval for the study was granted from the Human Research Ethics Board of the University of Alberta (Pro00138190). All eligible participants will be informed about the study, including its objective, potential risks and benefits, steps, the nature of their involvement, and their rights, such as the option to withdraw from the study at any time. Implied consent will be obtained from survey respondents, while written consent will be required from focus groups and priority-setting workshop participants prior to data collection. For eligible patients invited to participate in the study in clinical settings, consent to be contacted will be sought. Clinical data will be used exclusively to identify eligible participants, and identifiers will not be collected. Only members of the research team and the steering committee will have access to the aggregated data. Focus group participants will be reminded to keep shared views confidential, though complete confidentiality cannot be guaranteed in focus groups. A trained facilitator will ensure that all focus group participants have an equal opportunity to share their views and concerns. The demographic and salient characteristics of all stakeholder groups will be monitored to ensure representativeness, especially among vulnerable individuals within the patient group.

### Dissemination

The dissemination plan aims to raise awareness among various target audiences about research priorities for engagement in SPC that reflect the interests of both patients and care providers. These audiences will include patient representatives and advocacy groups, local grassroots and professional organizations, funders, and researchers. The top ten research priorities will be integrated into infographics and disseminated via email to all survey, focus group, and workshop participants, members of the steering committee, and target patient representatives and advocacy groups. Meetings with advocacy groups will be held to explore actionable strategies intended to influence public and private funders’ priorities regarding periodontal research, particularly the engagement of vulnerable groups in periodontal services. Social media will also be used to communicate the identified research priorities to the public.

A brief report summarizing the study findings will be shared with partnering grassroots agencies, professional organizations (e.g., Alberta Academy of Periodontists), and dental academic and non-academic clinics that supported the recruitment of study participants. These partners will also play a pivotal role in designing and implementing future research projects to address the identified research priorities. The brief report will also be shared with local and nationwide funding agencies supporting health research in Canada (e.g., Canadian Institutes of Health Research). Meetings and presentations will be held with target funders to disseminate the identified research priorities and explore ways of integrating these priorities into their financial support for oral health service research. The study results will be presented at both national (e.g., Canadian Academy of Periodontology annual meeting) and international (e.g., American Academy of Periodontology annual meeting) conferences. They will also be published in high-impact, peer-reviewed journals in the fields of periodontology (e.g., Journal of Periodontology) and dentistry in general (e.g., Journal of Dental Research) to reach academic and professional audiences.

Evaluation criteria for the dissemination plan will include the number and outputs of meetings held with advocacy groups, grassroots and professional organizations, and funders; the number of collaborations and partnerships formed with these partners; the uptake of the research priorities by relevant academic institutions and funding bodies; and the number of abstracts, oral presentations, and publications derived from the study.

## Discussion

The proposed study aims to identify the top ten research priorities or uncertainties for patient engagement in SPC that are most important to patients and care providers. Identified priorities are expected to inform and enhance the relevance of future research agendas for clinical practice. Research priorities have been traditionally determined by researchers and funding organizations [45]. While interests other than addressing pressing issues for healthcare stakeholders (e.g., scientific, political, commercial) may influence their priorities [46], the involvement of these stakeholders in defining research priorities can enhance the quality, relevance, and implementation of research projects, as well as researchers’ understanding of study phenomena and decisional contexts [47–49].

In conformity with the JLA approach [27], most research identifying research priorities in partnership with patients and care providers has focused on health conditions and treatments [28]. Although this emphasis theoretically includes disease prevention, screening, diagnosis, management, and health service utilization, patients’ uncertainties reported in previous studies have mainly been related to psychosocial issues, symptoms, and their consequences [28]. In contrast, care providers’ uncertainties have centered around treatment effectiveness [28]. Identified research priorities concerning patient engagement and the utilization of health services have mainly focused on strategies to improve accessibility, especially among vulnerable groups [33,50,51]. These findings suggest that patients’ and care providers’ uncertainties and concerns regarding patient engagement in health services have been insufficiently explored, identified, and prioritized.

Our study will address this gap by directly exploring uncertainties related to patient engagement in SPC. By investigating these uncertainties in the context of a chronic condition, our findings may be relevant to the management of other chronic diseases that require similar types and levels of patient engagement in specialized services. For instance, our results could be applicable to specialized services for chronic conditions where patients require referrals, engage in multiple phases (e.g., assessment, active treatment, and maintenance), and are expected to adhere to appointment schedules and treatment recommendations.

### Study strengths

Several strengths can be attributed to the study design. First, the study design follows a well-established approach that offers transparency and replicability for setting research priorities in partnership, where the uncertainties suggested by all the stakeholders are equally considered [27]. Second, qualitative and quantitative data will be used to identify and prioritize unanswered uncertainties regarding patient engagement in SPC. The qualitative data will also help describe pressing issues and concerns for patients and care providers regarding the study topic. Third, unlike many previous studies that either were not informed by literature reviews or lacked details on how the literature was searched [52], in our study, a comprehensive scoping review, including a quality assessment of the selected studies, will be conducted to verify whether the suggested uncertainties had already been addressed. While quality assessment is not required in scoping review studies, it appears to be necessary for judging whether the existing evidence is sufficient and credible to answer the suggested uncertainties. Fourth, three groups of periodontal care providers (general dentists, periodontists, and dental hygienists) will be involved to ensure the representation of care providers delivering this care. Additionally, actions will be taken to monitor and ensure the representativeness of patients, especially those from traditionally underserved populations and groups (e.g., senior, low-income individuals). Lastly, strong partnerships were developed with professional organizations, academic and non-academic dental clinics, and patient representatives to enhance the recruitment of participants and facilitate knowledge dissemination upon completion of the study.

### Study limitations

Our study also has some limitations that need to be acknowledged. First, it will involve only patients and care providers from the province of Alberta, Canada. Therefore, the generalizability of the study findings will depend on the similarities between the study context and implementation contexts, including target populations. It is important to keep in mind that involving participants from different contexts and jurisdictions is not without consequences, as issues related to patient engagement in health services tend to be context-specific [42]. Second, the recruitment of participants may be subject to selection bias, as those who choose to participate, whether by completing a survey, attending a focus group, or participating in the prioritization workshop, may not fully represent the stakeholder groups involved in the study. Research suggests that the involvement of patients and caregivers from vulnerable groups (e.g., ethnic minorities) in setting research priorities may be challenging [53]. To minimize this bias, several recruitment strategies will be employed, and the demographic and other salient characteristics (e.g., clinical experience) of participants will be monitored to ensure the representativeness of all stakeholder groups. Third, the use of online surveys to collect uncertainties may introduce information bias, as it likely favors individuals who have access to and are familiar with the Internet [54]. To address this bias, alternative methods for survey participation will be considered, such as paper-based surveys, telephone interviews, or in-person interviews. Lastly, the level of participation and the number of suggested uncertainties may vary across stakeholder groups, depending on their familiarity with research, the breadth and depth of their experiences related to patient engagement in SPC, and their ability to identify and articulate uncertainties that can be addressed through research. This issue will be addressed by employing various methods (e.g., focus groups, individual interviews, surveys) to accommodate their preferences and communication styles.

### Future research

An important future direction is to facilitate the uptake of the identified research priorities by both researchers and funders, a challenge that has been consistently observed in studies involving partnership-based priority setting [30]. The involvement of patients and care providers in setting research priorities is an important step; however, it does not automatically ensure that the identified priorities will be addressed through research or that they will ultimately improve clinical practice. It has been observed that priority-setting exercises often lack rigorous evaluation of their utility, while some evidence suggests there are still mismatches between the research priorities identified by stakeholders, the research agendas being pursued, and the resource allocations by public and private funding bodies after these exercises [42,45,55]. Several strategies have been suggested to improve the uptake of identified research priorities in partnership [27,55], some of which are summarized in Table 3. The findings of the present study will be purposefully and strategically disseminated to oral health researchers and funders to enhance the uptake of the resulting research priorities and will inform the research agenda of our research team, specifically the ongoing research program on patient engagement in SPS in both academic and non-academic dental institutions in Canada.

**Table 3:** Strategies to improve the uptake of uncertainties by researchers and funding bodies.

*Build partnerships*: Establish strong, long-lasting partnerships with funding agencies for dissemination purposes, and with local organizations to facilitate the implementation of research projects addressing the identified uncertainties. *Plan dissemination*: Develop a comprehensive plan to disseminate the top ten uncertainties. *Tailor dissemination*: Target research communities and funders based on the type of uncertainty. *Translate uncertainties*: Translate the identified uncertainties into well-defined research questions, specifying the most suitable methods to address them. *Communicate effectively*: Select individuals who can effectively communicate the top ten uncertainties to researchers and funders, presenting them as relevant research questions and topics. *Evaluate effectiveness*: Conduct a thorough evaluation of the dissemination plan to assess whether the dissemination goals have been achieved and make adjustments if necessary. *Self-assess alignments*: Encourage researchers and funders to assess the extent to which their research priorities align with those of healthcare stakeholders and act accordingly. *Formally assess alignments*: Conduct research aiming to rigorously assess whether the research priorities of researchers and funders match those of healthcare stakeholders.

## Conclusions

The application of the JLA approach to setting research priorities in partnership is expected to effectively identify and prioritize the unanswered uncertainties that matter most to patients and care providers regarding patient engagement in SPC in Alberta, Canada. Concerted efforts will be necessary to improve the uptake of the resulting research priorities by oral health researchers and funders. The study findings are also expected to demonstrate the effectiveness and feasibility of the chosen approach in developing stakeholder-led research priorities in other health disciplines (e.g., dentistry) and issues (e.g., patient engagement in health services).

## References

[1] Kassebaum NJ, Bernabé E, Dahiya M, Bhandari B, Murray CJL, Marcenes W. Global burden of severe periodontitis in 1990-2010: a systematic review and meta-regression. J Dent Res. 2014;93(11):1045–53. doi: 10.1177/0022034514552491 25261053 PMC4293771

[2] Eke PI, Thornton-Evans GO, Wei L, Borgnakke WS, Dye BA, Genco RJ. Periodontitis in US Adults: National Health and Nutrition Examination Survey 2009-2014. J Am Dent Assoc. 2018;149(7):576–588.e6. doi: 10.1016/j.adaj.2018.04.023 29957185 PMC8094373

[3] World Health Organization. Oral health Court Profile. Retrieved from https://www.who.int/publications/m/item/oral-health-can-2022-country-profile

[4] Tonetti MS, Jepsen S, Jin L, Otomo-Corgel J. Impact of the global burden of periodontal diseases on health, nutrition and wellbeing of mankind: A call for global action. J Clin Periodontol. 2017;44(5):456–62. doi: 10.1111/jcpe.12732 28419559

[5] Buset SL, Walter C, Friedmann A, Weiger R, Borgnakke WS, Zitzmann NU. Are periodontal diseases really silent? A systematic review of their effect on quality of life. J Clin Periodontol. 2016;43(4):333–44. doi: 10.1111/jcpe.12517 26810308

[6] Borges T de F, Regalo SC, Taba M Jr, Siéssere S, Mestriner W Jr, Semprini M. Changes in masticatory performance and quality of life in individuals with chronic periodontitis. J Periodontol. 2013;84(3):325–31. doi: 10.1902/jop.2012.120069 22548588

[7] Beck J, Papapanou P, Philips K, Offenbacher S. Periodontal medicine: 100 years of progress. J Dent Res. 2019;98(10):1053–62. 31429666 10.1177/0022034519846113

[8] Economist Intelligence Unit. 2021. Time to take gum disease seriously: The societal and economic impact of periodontitis. Retrieved from https://impact.economist.com/perspectives/sites/default/files/eiuefp-oralb-gum-disease.pdf

[9] Henshaw MM, Garcia RI, Weintraub JA. Oral Health Disparities Across the Life Span. Dent Clin North Am. 2018;62(2):177–93. doi: 10.1016/j.cden.2017.12.001 29478452

[10] Chávez EM, Wong LM, Subar P, Young DA, Wong A. Dental Care for Geriatric and Special Needs Populations. Dent Clin North Am. 2018;62(2):245–67. doi: 10.1016/j.cden.2017.11.005 29478456

[11] Huang J, Yao Y, Jiang J, Li C. Effects of motivational methods on oral hygiene of orthodontic patients: A systematic review and meta-analysis. Medicine (Baltimore). 2018;97(47):e13182. doi: 10.1097/MD.0000000000013182 30461616 PMC6392669

[12] Liaw A, Miller C, Nimmo A. Comparing the periodontal tissue response to non-surgical scaling and root planing alone, adjunctive azithromycin, or adjunctive amoxicillin plus metronidazole in generalized chronic moderate-to-severe periodontitis: a preliminary randomized controlled trial. Aust Dent J. 2019;64:145–52. 30628088 10.1111/adj.12674

[13] Teughels W, Feres M, Oud V, Martín C, Matesanz P, Herrera D. Adjunctive effect of systemic antimicrobials in periodontitis therapy: A systematic review and meta-analysis. J Clin Periodontol. 2020;47 Suppl 22:257–81. doi: 10.1111/jcpe.13264 31994207

[14] Wang H-L, Greenwell H, Fiorellini J, Giannobile W, Offenbacher S, Salkin L, et al. Periodontal regeneration. J Periodontol. 2005;76(9):1601–22. doi: 10.1902/jop.2005.76.9.1601 16171453

[15] Wong LB, Yap AU, Allen PF. Periodontal disease and quality of life: Umbrella review of systematic reviews. J Periodontal Res. 2021;56(1):1–17. doi: 10.1111/jre.12805 32965050

[16] Marín-Jaramillo R, Agudelo-Suárez A. Factors related to compliance with periodontal disease treatment appointments: A literature review. J Clin Exp Dent. 2022;14(11):e967–71. 36458036 10.4317/jced.59752 PMC9701343

[17] Clancy CM. Patient engagement in health care. Health Serv Res. 2011;46(2):389–93. doi: 10.1111/j.1475-6773.2011.01254.x 21371026 PMC3064908

[18] Doyle C, Lennox L, Bell D. A systematic review of evidence on the links between patient experience and clinical safety and effectiveness. BMJ Open. 2013;3(1):e001570. doi: 10.1136/bmjopen-2012-001570 23293244 PMC3549241

[19] Barello S, Graffigna G, Vegni E. Patient engagement as an emerging challenge for healthcare services: mapping the literature. Nurs Res Pract. 2012;2012:905934. 23213497 10.1155/2012/905934 PMC3504449

[20] Wennström JL, Serino G, Lindhe J, Eneroth L, Tollskog G. Periodontal conditions of adult regular dental care attendants. A 12-year longitudinal study. J Clin Periodontol. 1993;20(10):714–22. doi: 10.1111/j.1600-051x.1993.tb00696.x 8276981

[21] Sonnenschein SK, Kohnen R, Ruetters M, Krisam J, Kim T-S. Adherence to long-term supportive periodontal therapy in groups with different periodontal risk profiles. J Clin Periodontol. 2020;47(3):351–61. doi: 10.1111/jcpe.13252 31912538

[22] Amerio E, Mainas G, Petrova D, Giner Tarrida L, Nart J, Monje A. Compliance with supportive periodontal/peri-implant therapy: A systematic review. J Clin Periodontol. 2020;47(1):81–100. doi: 10.1111/jcpe.13204 31562778

[23] Lee J, Bennett D, Richards P, Inglehart M. Periodontal referral patterns of general dentists: lessons for dental education. J Dent Educ. 2009;73(2):199–210. 19234076

[24] Kraatz J, Hoang H, Ivanovski S, Crocombe LA. Non-Clinical Factors Associated With Referrals to Periodontal Specialists: A Systematic Review. J Periodontol. 2017;88(1):89–99. doi: 10.1902/jop.2016.160318 27452395

[25] Perrell-Jones C, Ireland RS. What factors influence patient compliance with supportive periodontal therapy in a general practice setting?. Br Dent J. 2016;221(11):701–4. doi: 10.1038/sj.bdj.2016.904 27932844

[26] Delatola C, Adonogianaki E, Ioannidou E. Non-surgical and supportive periodontal therapy: predictors of compliance. J Clin Periodontol. 2014;41(8):791–6. doi: 10.1111/jcpe.12271 24813661 PMC4469950

[27] National Institute for Health Research. 2018. The James Lind Alliance guidebook: Version 7. Available at: http://www.jla.nihr.ac.uk/jlaguidebook/downloads/Print-JLA-guidebook-version-7-March-2018.pdf

[28] Nygaard A, Halvorsrud L, Linnerud S, Grov EK, Bergland A. The James Lind Alliance process approach: scoping review. BMJ Open. 2019;9(8):e027473. doi: 10.1136/bmjopen-2018-027473 31473612 PMC6720333

[29] Wilkins CH, Miller ST, Richmond AN, Carrasquillo O. Community-Engaged Research - Essential to Addressing Health Inequities. N Engl J Med. 2023;389(21):1928-1931. 37982404 10.1056/NEJMp2307774 PMC11088953

[30] Finer S, Robb P, Cowan K, Daly A, Shah K, Farmer A. Setting the top 10 research priorities to improve the health of people with Type 2 diabetes: a Diabetes UK-James Lind Alliance Priority Setting Partnership. Diabet Med. 2018;35(7):862–70. doi: 10.1111/dme.13613 29485717 PMC6032840

[31] Kelly S, Lafortune L, Hart N, Cowan K, Fenton M, Brayne C, et al. Dementia priority setting partnership with the James Lind Alliance: using patient and public involvement and the evidence base to inform the research agenda. Age Ageing. 2015;44(6):985–93. doi: 10.1093/ageing/afv143 26504119 PMC4621237

[32] Deane KHO, Flaherty H, Daley DJ, Pascoe R, Penhale B, Clarke CE, et al. Priority setting partnership to identify the top 10 research priorities for the management of Parkinson’s disease. BMJ Open. 2014;4(12):e006434. doi: 10.1136/bmjopen-2014-006434 25500772 PMC4281559

[33] Hemmelgarn BR, Pannu N, Ahmed SB, Elliott MJ, Tam-Tham H, Lillie E, et al. Determining the research priorities for patients with chronic kidney disease not on dialysis. Nephrol Dial Transplant. 2017;32(5):847–54. doi: 10.1093/ndt/gfw065 27190349 PMC5837625

[34] van Furth EF, van der Meer A, Cowan K. Top 10 research priorities for eating disorders. Lancet Psychiatry. 2016;3(8):706–7. doi: 10.1016/S2215-0366(16)30147-X 27475763

[35] Lechelt LA, Rieger JM, Cowan K, Debenham BJ, Krewski B, Nayar S, et al. Top 10 research priorities in head and neck cancer: Results of an Alberta priority setting partnership of patients, caregivers, family members, and clinicians. Head Neck. 2018;40(3):544–54. doi: 10.1002/hed.24998 29149525

[36] Fitzcharles M-A, Brachaniec M, Cooper L, Dubin R, Flynn T, Gerhold K, et al. A paradigm change to inform fibromyalgia research priorities by engaging patients and health care professionals. Can J Pain. 2017;1(1):137–47. doi: 10.1080/24740527.2017.1374820 35005349 PMC8730558

[37] Krueger R. Developing Questions for Focus Groups. SAGE Publications; 1998.

[38] Wells G, Shea B, O’Connell D, Peterson J, Welch V, Losos M, et al. The Newcastle-Ottawa Scale (NOS) for assessing the quality of nonrandomised studies in meta-analyses [Internet]. Ottawa: Ottawa Hospital Research Institute; 2008 [cited 2024 Jan]. Available from: http://www.ohri.ca/programs/clinical_epidemiology/oxford.asp

[39] Arksey H, O’Malley L. Scoping studies: Towards a methodological framework. International Journal of Social Research Methodology. 2005;8(1):19–32.

[40] Tricco AC, Lillie E, Zarin W, O’Brien KK, Colquhoun H, Levac D, et al. PRISMA Extension for Scoping Reviews (PRISMA-ScR): Checklist and Explanation. Ann Intern Med. 2018;169(7):467–73. doi: 10.7326/M18-0850 30178033

[41] Taylor CJ, Huntley AL, Burden J, Gadoud A, Gronlund T, Jones NR, et al. Research priorities in advanced heart failure: James Lind alliance priority setting partnership. Open Heart. 2020;7(1):e001258. doi: 10.1136/openhrt-2020-001258 32606070 PMC7328807

[42] Knight SR, Metcalfe L, O’Donoghue K, Ball ST, Beale A, Beale W, et al. Defining Priorities for Future Research: Results of the UK Kidney Transplant Priority Setting Partnership. PLoS One. 2016;11(10):e0162136. doi: 10.1371/journal.pone.0162136 27776143 PMC5077146

[43] Gallagher M, Hares T, Spencer J, Bradshaw C, Webb I. The nominal group technique: A research tool for general practice?. Family Practice. 1993;10:76–81. 8477899 10.1093/fampra/10.1.76

[44] Elo S, Kyngäs H. The qualitative content analysis process. J Adv Nurs. 2008;62(1):107–15. doi: 10.1111/j.1365-2648.2007.04569.x 18352969

[45] Levelink M, Voigt-Barbarowicz M, Brütt AL. Priorities of patients, caregivers and health-care professionals for health research - A systematic review. Health Expect. 2020;23(5):992–1006. doi: 10.1111/hex.13090 32643854 PMC7696132

[46] Chalmers I, Bracken MB, Djulbegovic B, Garattini S, Grant J, Gülmezoglu AM, et al. How to increase value and reduce waste when research priorities are set. Lancet. 2014;383(9912):156–65. doi: 10.1016/S0140-6736(13)62229-1 24411644

[47] van Middendorp JJ, Allison HC, Ahuja S, Bracher D, Dyson C, Fairbank J, et al. Top ten research priorities for spinal cord injury: the methodology and results of a British priority setting partnership. Spinal Cord. 2016;54(5):341–6. doi: 10.1038/sc.2015.199 26554273 PMC5399156

[48] Price A, Albarqouni L, Kirkpatrick J, Clarke M, Liew SM, Roberts N, et al. Patient and public involvement in the design of clinical trials: An overview of systematic reviews. J Eval Clin Pract. 2018;24(1):240–53. doi: 10.1111/jep.12805 29076631

[49] Pollock A, St George B, Fenton M, Firkins L. Top 10 research priorities relating to life after stroke--consensus from stroke survivors, caregivers, and health professionals. Int J Stroke. 2014;9(3):313–20. doi: 10.1111/j.1747-4949.2012.00942.x 23227818

[50] Aldiss S, Hart-Spencer P, Langton L, Malik S, McEvoy K, Morgan JE, et al. What matters to you? Engaging with children in the James Lind Alliance Children’s Cancer Priority Setting Partnership. Res Involv Engagem. 2023;9(1):110. doi: 10.1186/s40900-023-00518-2 38037183 PMC10688066

[51] Finlay-Jones A, Sampson R, Parkinson A, Prentice K, Bebbington K, Treadgold C, et al. Priority setting for children and young people with chronic conditions and disabilities. Health Expect. 2023;26(4):1562–74. doi: 10.1111/hex.13761 37078632 PMC10349250

[52] Bourne AM, Johnston RV, Cyril S, Briggs AM, Clavisi O, Duque G, et al. Scoping review of priority setting of research topics for musculoskeletal conditions. BMJ Open. 2018;8(12):e023962. doi: 10.1136/bmjopen-2018-023962 30559158 PMC6303563

[53] Wan YL, Beverley-Stevenson R, Carlisle D, Clarke S, Edmondson RJ, Glover S, et al. Working together to shape the endometrial cancer research agenda: The top ten unanswered research questions. Gynecol Oncol. 2016;143(2):287–93. doi: 10.1016/j.ygyno.2016.08.333 27593736

[54] Lough K, Hagen S, McClurg D, Pollock A, JLA Pessary PSP Steering Group. Shared research priorities for pessary use in women with prolapse: results from a James Lind Alliance Priority Setting Partnership. BMJ Open. 2018;8(4):e021276. doi: 10.1136/bmjopen-2017-021276 29705767 PMC5931298

[55] Crowe S, Fenton M, Hall M, Cowan K, Chalmers I. Patients’, clinicians’ and the research communities’ priorities for treatment research: there is an important mismatch. Res Involv Engagem. 2015;1:2. doi: 10.1186/s40900-015-0003-x 29062491 PMC5598091

